# Anatomical, Histological, and Morphometrical Features of the Auditory Ossicles in Dog Fetuses at 55–56 Days of Gestation

**DOI:** 10.3390/vetsci13070624

**Published:** 2026-06-27

**Authors:** Cristian-Olimpiu Martonos, Cristian Constantin Dezdrobitu, Little William Brady, Laszlo-Andras Nagy, David Hilchie, Pompei Bolfa, Alexandru Ion Gudea

**Affiliations:** 1 Department of Biomedical Sciences, Ross University School of Veterinary Medicine, Basseterre P.O. Box 334, Saint Kitts and Nevis; cmartonos@rossvet.edu.kn (C.-O.M.); cdezdrobitu@rossvet.edu.kn (C.C.D.); brlittle@rossvet.edu.kn (L.W.B.); anagy@rossvet.edu.kn (L.-A.N.); dhilchie@rossvet.edu.kn (D.H.); pompeibolfa@gmail.com (P.B.); 2 Department of Anatomy, Faculty of Veterinary Medicine, University of Agricultural Sciences and Veterinary Medicine, 400372 Cluj-Napoca, Romania

**Keywords:** auditory ossicles, incus, malleus, stapes, histology, morphometry, indices

## Abstract

This study examines the auditory ossicles of 55–56-day gestational age dog fetuses and shows that the basic three-bone middle ear pattern is already present, but the bones remain partly cartilaginous and are actively ossifying. The tympanic cavity contained a gelatinous, mesenchyme-like material, making dissection difficult. The malleus was the largest and most laterally located ossicle, with an oval head, short neck, curved manubrium, and notable variability in the anterior process. The incus had a rectangular body with short and long processes of similar length, and the lenticular process plus a synovial incudo-malleal joint were already present. The stapes was the smallest ossicle, triangular in shape, with a shorter posterior crus and early ossification in the distal crura and footplate. Histology showed active bone formation with medullary cavities, trabeculae, and osteoclast activity in specific regions, especially the malleal neck, the incudal long process, and the distal stapes, as the metrical data may reflect early mechanical specialization. Overall, the paper concludes that fetal dogs already possess the mammalian ossicular plan, but the structures remain immature, reflecting a clear histological evolution from cartilage-dominant tissue toward organized bone.

## Simple Summary

This study describes the auditory ossicles of 55–56-day (gestational age) dog fetuses using anatomical, histological, and morphometric methods. The fetal middle ear already contains the malleus, incus, and stapes, but they are still partly cartilaginous and actively ossifying. The malleus is the largest ossicle and shows a curved manubrium and a variable anterior process. The incus has a rectangular body with short and long crura and a present lenticular process. The stapes is the smallest, triangular ossicle, with ossification beginning in the crura and footplate. Histology shows medullary cavities, trabeculae, and osteoclast activity, indicating ongoing bone remodeling. The authors conclude that the basic mammalian three-ossicle pattern is already established in fetal dogs, but the structures continue to mature after this stage.

## 1. Introduction

As our closest animal companion, domestic dogs have a controversial history of global distribution and evolution [1]. The history of domestic dogs (*Canis lupus familiaris*) dates back approximately 33,000 years, when their ancestors split from grey wolves, somewhere in southern East Asia [2]. Domesticated dogs arrived in Europe about 10,000 years ago, and a Siberian line of dogs seems to be the most likely ancestor of American dogs [1,3].

The ear (*organum vestibulocochleare*) is a sense organ that hosts the senses of hearing and balance and consists of three segments: the external ear (*auris externa*), the middle ear (*auris media*), and the inner ear (*auris interna*) [4,5,6,7]. The most lateral segment, the external ear, receives the sound waves; the middle ear transmits the vibrations to the most medial segment, the internal ear, where they are converted into electrical signals [8]. It is an example of a perfect mechanism for transmitting air vibrations produced by sounds to the fluid-filled inner ear compartment called the cochlea (*cochlea*) [9,10].

The middle compartment of the vestibulocochlear organ of mammals contains three auditory ossicles, the malleus (*malleus*), the incus (*incus*), and the stapes (*stapes*), as well as their adjacent ligaments and muscles [10,11,12]. Although a single stapes, known as columella, alone is well documented in mammalian ancestors [12], reptiles and birds [9,13], one of the distinguishing features of extant mammals is the presence of all three auditory ossicles [13], which bridge the gap between the tympanic membrane (*membrana tympani*) and the vestibular fenestra (*fenestra vestibuli*). This type of three-ossicle middle ear is better adapted for higher-frequency sounds and allows some mammals to even perceive ultrasonic frequencies [9]. Particularly adapted characteristics have been reported in chinchillas, guinea pigs, and a few other rodents, in which the first two auditory ossicles form a single fused structure [6,7,14]. The macroscopic and functional features of the mammalian middle ear allow for the categorization of several functional types: monotreme ears; therian ancestral ears; microtype ears; transitional ears; freely mobile ears; ctenohystrica ears; and cetacean ears [6,7,10,15,16].

Anatomically, the aerated segment located medially to the tympanic membrane can be divided into five compartments (mesotympanum, epitympanum, hypotympanum, protympanum, and retrotympanum) and has six walls [10].

The earliest morphological description of the auditory ossicles in carnivores was provided by A. H. G. Doran in 1879 in his text while the first experiments testing the audibility of shrill notes were reported by F. Galton [17,18]. Recent studies have reported a negative correlation between body size and the interaural width of terrestrial mammals, as well as their capacity to respond to high-frequency sounds [18,19,20]. It is also well recognized that the size, shape, and stiffness of the auditory ossicles play an important role in sound transmission [21,22,23].

Although the postnatal features of the auditory ossicles are well documented, the anatomical characteristics of the ossicles during the fetal period are poorly documented in many species. The current literature contains only a few articles that describe the morphological aspects of the fetal auditory ossicles in humans [4,4,24,25,26,27] and a small number that describe the fetal stage of these ossicles in the veterinary field [6,14,28,29].

The present study aims to fill this gap by providing an anatomical, histological, and morphometric characterization of the auditory ossicles in dog fetuses at 55–56 days of gestation and comparing the results with previously reported data for adult canines.

## 2. Materials and Methods

### 2.1. Origin of the Biological Material and Image Processing Technique

The biological material utilized for research was provided in accordance with the 3Rs paradigm. It consisted of fetal canine tissues obtained from pregnant female cadavers attained for the Gross Anatomy course at Ross University School of Veterinary Medicine, Saint Kitts and Nevis. These tissues were obtained through the routine supply chain established between the University and a commercial supplier in the United States (USDA AHPIS export approval and a Caribbean Authority import permit). The material is consistently stored in freezers and used for teaching. The fetuses have been collected during the academic year 2024–2025. Six canine fetuses, three males and three females, with approximately the same gestational age, were selected for the present study. The cadavers were of mid-sized, mixed breed females of approximately 20 kg.

To avoid misinterpretation of gestational age, the female specimens were initially categorized into three groups: small (<9 kg), medium (9–40 kg), and large (>40 kg), as previously reported. This study used mathematical formulas to estimate gestational age accurately. Two standard formulas were used to assure accurate determination of the gestational age (GA) where GA = (3 × CRL) + 27, where 3 and 27 are constant values, and CRL is the crown-rump length (cm) of the fetus and the second formula utilized was GA = (15 × HD) + 20, where 15 and 20 are constant values and HD is the dead diameter measured in the parietal area (biparietal diameter) [30,31,32].

This research attained access to the temporal area by following previously reported procedures for opening the tympanic cavity. First, a ventral incision was made with entry into the ventral wall of the tympanic bulla via sharp dissection tools, providing access to the middle ear’s internal structures. To ensure that the biological debris had been removed, a gentle lavage with normal saline solution was performed. Next, the ossicular chain could be removed, and the ossicles could be visually identified. The auditory ossicles were preserved in formaldehyde for 14 days.

For accurate visualization and digital image acquisition of the samples, an Olympus SZX7 dissecting microscope with an incorporated DP27 camera was used. The selected images were enhanced in Adobe Photoshop^®^ (San Jose, CA, USA). For the morphometric data, ImageJ 1.46^®^ was utilized to perform measurements [33].

### 2.2. Histological Technique

Following previously reported techniques, the auditory ossicles collected from the 66-day-old fetuses (individuals A and D) were fixed in 10% neutral buffered formalin for 14 days [34] and then immersed in RDO Rapid Decalcifier (Apex Engineering Products Corporation, USA) solution for one hour. After the washing process, the ossicles were placed in a Tissue-Tek E-300 processor (Rankin Biomedical Corporation, Davisburgh, MIUSA^®^) for overnight processing. Samples were cut at 4 µm using a Leica RM 2155 microtome (Leica Microsystems GmBh^®^, Nussloch Germany ) with Tissue-Tek Feather 4980 low-profile blades (Sakura Finetek USA, Inc^®^., Torrance). The Harris hematoxylin and eosin (H&E) protocol was used to stain the samples [34]. An Olympus light microscope and Olympus DP26 digital camera (Olympus Surgical Technologies America^®^), utilizing CellSens 4.1 Standard image analysis software (Evident Scientific^®^), were used to examine and assess the slides.

### 2.3. Morphometric Aspects and Measurements

Measurements were obtained using a standardized, widely accepted systematic system (Table 1, Table 2 and Table 3), as previously described in otology studies of humans and other primates and mammals (Table 1, Table 2 and Table 3) [35].

All measurements were taken by one operator using the same method and calibration in ImageJ^®^, with the same scale. All photographs were taken by a different operator (to reduce biases). Measurements were attained from a series of images and at different angles. This approach made some measurements impossible due to the tissue’s innate presentation and minute size. Data was recorded in a Google spreadsheet (allowing direct calculation of indices).

Data was recorded for each individual separately. Basic statistical processing was carried out using Google Sheets’ simple statistics feature, the XL Miner Analysis Toolpack add-on 2023 (FrontlineSolvers—www.solver.com (accessed on 14 May 2026).

## 3. Results

The most important feature observed immediately after the tympanic bulla was opened was the presence of a gelatinous substance filling the tympanic cavity, which embedded the auditory ossicles and the adjacent ligaments and muscles. This gelatinous fluid invaded all segments of the middle ear (*auris media*), including the epitympanic recess, the tympanic cavity proper, and the tympanic bulla. The presence of this substance, in combination with the predominantly cartilaginous structure of the auditory ossicles, made the extraction of the auditory ossicles in dog fetuses at 55–56 days of gestation relatively difficult.

The auditory ossicles were located in the middle segment of the vestibulocochlear organ (*organum vestibulocochleare*) and bridged the gap between the tympanic membrane laterally and the vestibular fenestra medially.

From lateral to medial, the *ossicula auditus* allowed identification of three structures: the malleus (*malleus*), the incus (*incus*), and the stapes (*stapes*).

**Figure 1:**
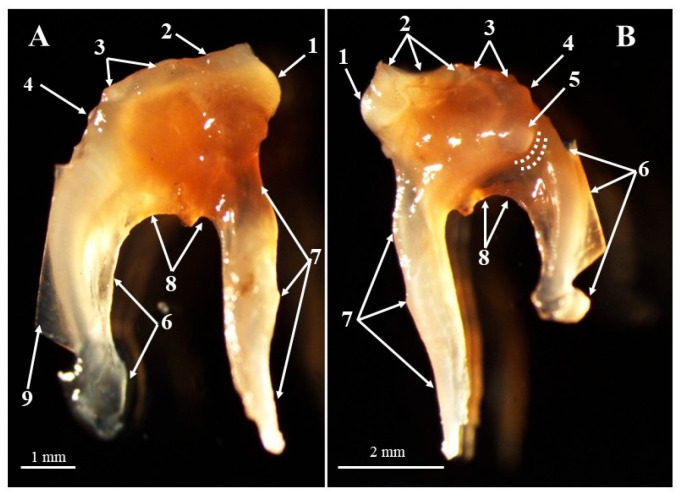
Malleus—anatomic features. (**A**)—lateral view; (**B**)—medial view. 1. Head of the malleus; 2. articular surface; 3. neck of the malleus; 4. lateral process; 5. muscular process; 6. handle of the malleus; 7. anterior process; 8. bony lama; 9. tympanic membrane. Dotted lines—groove of the chorda tympany nerve.

In these specimens, the malleus (Figure 1) was the largest auditory bone and was most laterally positioned. The morphological investigation allowed identification of three significant regions of the malleus: the head of the malleus (*caput mallei*), the neck of the malleus (*collum mallei*), and the handle of the malleus (*manubrium mallei*) (Figure 1). The head of the malleus was small with an oval shape and was accommodated by the most dorsal segment of the middle ear, the epitympanic recess. On its caudo-medial side, the articular surface was noted, which forms the incudomallear joint (*articulatio incudomallearis*) together with the incus. Laterally, the head of the malleus is contiguous with the neck of the malleus. This short segment (Table 4), running in a lateromedial direction, connects the head of the malleus to its handle. Anatomical characteristics allow identification of three distinct processes in this area: the rostral process *(processus rostralis*), the muscular process (*processus muscularis*), and the lateral process (*processus lateralis*) (Figure 1).

The anterior process was located on the ventral aspect of the neck of the malleus, ventral to the malleal head. This process exhibited a wide range of lengths (Table 4). From its base, the process was oriented ventrolaterally and covered by ligaments. In specimens with a relatively long anterior process, the process was even longer than the malleus handle and parallel to it. The ventral surface of the malleal neck, the proximomedial segment of the manubrium, and the base of the anterior process were connected via a fine bony lamina.

The distal extremity of the medial side of the malleal neck bears the muscular process of the malleus. Occurring as a conical projection on the medial side of the malleus, this structure forms the insertion point for the tensor tympani muscle (*m. tensor tympani*). Its tip faced medially in all studied specimens, perpendicular to the longitudinal axis of the manubrium. Immediately ventro-caudal by the base of the muscular process, the medial surface of the neck of the malleus was marked by a shallow groove which accommodated the chorda tympani nerve (*chorda tympani*).

The lateral process was the smallest and marked the distal end of the middle segment of the malleus. This part does not come in contact with the inner side of the tympanic membrane.

The *manubrium mallei* segment continued laterally from the malleus, and its lateral surface came into contact with the internal surface of the tympanic membrane. Although its proximal end had an obvious triangular cross-section, its distal end appeared as a double-sided spatulate structure- the “banana-shaped” *manubrium mallei*.

**Figure 2:**
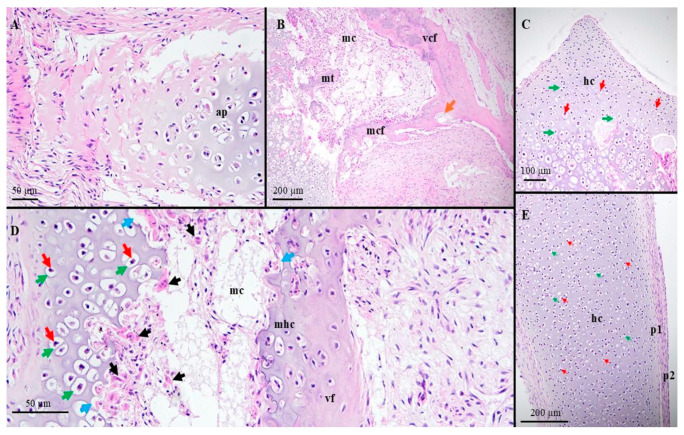
Histological features of the malleus in dog fetuses at 55–56 days of gestation. (**A**) anterior process/H&E staining; (**B**) malleal head area/H&E staining; (**C**) malleal head/H&E staining; (**D**) malleal head, articular area/H&E staining; (**E**) malleal handle ap—anterior process; mc—medullary cavity; mt—mineralized trabeculae; mcf—medial cortical fascicle; vfc—ventral cortical fascicle; mhc—mineralized hyaline cartilage; hc—hyaline cartilage; p1-perichondrium, chondrogenic layer; p2—perichondrium, fibrous layer; red arrows—chondrocyte; green arrows—condrocyte lacunae; black arrows—osteoclast; blue arrows—Howship’s lacunae; orange arrow—blood vessel.

Histologically, the malleus ossicle in dog fetuses at 55–56 days of gestation was mainly cartilaginous, with hyaline cartilage as the predominant tissue observed (Figure 2).

At this age of gestation, ossification had already begun forming two thin cortical fascicles (medial and ventral) in the neck region. Both fascicles originated at the base of the anterior process and have a divergent distribution. The medial one ran dorso-medially and connected the base of the anterior process with the malleal head, while the ventral was visualized in a lateroventral orientation and attached at the level of the proximal end of the manubrium.

Dorsal to the above-mentioned cortical fascicles, the malleal head and neck contained a large common medullary cavity populated with endochondral bony trabeculae and bone marrow with blood vessels (Figure 2). The peripheral area of the head of the malleus, the articular surface, and the dorsal segment of the neck of the malleus were associated with a thick hyaline cartilaginous layer. The peripheral area of the medullary cavity was infiltrated with a large number of osteoclasts, indicating that the remodeling of the bony tissue is ongoing at this early fetal age. The lateral process and the handle were cartilaginous in nature, surrounded by a double-layered perichondrium made up of an inner chondrogenic layer and an outer fibrous layer.

The anterior process of the malleus was connected via the medial cortical fascicle and intramembranous ossification (Figure 2).

**Figure 3:**
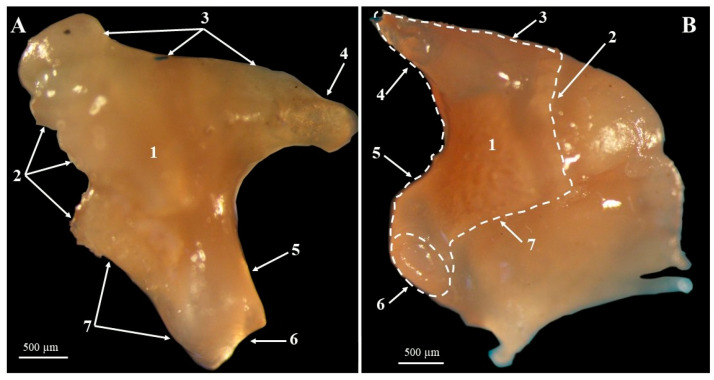
Incus—anatomical features. (**A**)—lateral view; (**B**)—medial view. 1. Body of incus; 2. articular surface; 3. dorsal margin of the body of the incus; 4. short process; 5. long process; 6. lenticular process; 7. Ventral margin of the body of the incus.

The *incus* (Figure 3) was noted as being in the intermediate position of the auditory chain, being margined laterally by the malleus and medially by the stapes. Topographically, it was situated at the level of the epitympanic recess and shares a small cavity with the most medial segment of the malleus. Overall, it presented with a rectangular body (*corpus incudis*) and two caudal processes (*crura*), resembling a biradicular molar tooth (Figure 3). The cranial segment of the incus was covered by an articular surface adjacent to the articular surface found on the caudo-medial aspect of the head of the malleus to create the incudomalleal joint.

The two crura of the incus exhibited as caudal continuations in divergent directions. Morphometrically, these two processes were similar in length (Table 5). The smaller process (*crus breve*) was continued dorso-caudally from the dorsal margin of the incus. Its terminal end displayed a conical appearance and was accommodated by a caudal diverticulum of the epitympanic recess. The attachments to the bony walls were provided by a few ligamentous structures (*ligg. incudis*).

The other larger process (*crus longus)* continued from the ventral margin of the incus (Table 5). The long process was oriented ventromedially and after a short distance a pronounced medial bend was observed. At the distal end of the long process a cartilaginous bulbous enlargement resembling the lenticular process (*processus lenticularis*) was observed as previously described in adult specimens. The presence of this cartilaginous lenticular process, along with the obvious curvature of the long process, could be used as a landmark to distinguish between the *crus brevis* and *crus longus* of the incus in these specimens.

**Figure 4:**
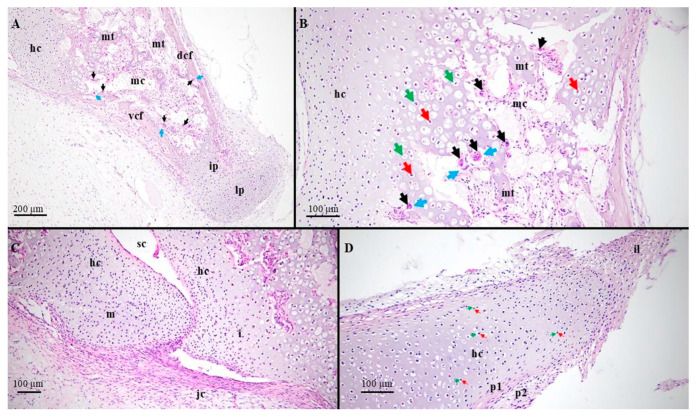
Histological features of the incus in dog fetuses at 55–56 days of gestation. (**A**) long process/H&E staining; (**B**) incus, body area/H&E staining; (**C**) incudomalleal joint/H&E staining; (**D**) short process/H&E staining; m—malleal head; i—incudal body; mc—medullary cavity; mt—mineralized trabeculae; ip—incudal pedicle; il—incudal ligament; lp—lenticular process; dcf—dorsal cortical fascicle; vfc—ventral cortical fascicle; hc—hyaline cartilage; sc—synovial cavity; jc—incudomalleal joint capsule; p1-perichondrium, chondrogenic layer; p2—perichondrium, fibrous layer; red arrows—chondrocyte; green arrows-condrocyte lacuna; black arrows—osteoclast; blue arrows—Howship’s lacunae.

The histological assessment of the incus revealed that the process of fetal ossification was active in the long process of the incus represented by two bony cortical fascicles (ventral and dorsal) which ceased distally near the incudal pedicle. The lenticular process in the dog fetuses at 55–56 days of gestation was totally cartilaginous (Figure 4).

Proximally, the ventral cortical fascicle projected from the ventral margin of the body of the incus while the dorsal cortical fascicle terminated before touching the base of the short process of the incus.

The incudal body possessed a cavitary feature with visible mineralized endosteal trabeculae. The medullary cavity exhibited a labyrinthine pattern, and many small blood vessels were identified. The segment of the body that corresponds to the incudomalleal articulation together with the adjacent areas maintained a cartilaginous structure at this gestational period (Figure 4).

The short process was totally cartilaginous, and its distal extremity bore the incudal ligament making up a component of the incudopetrosal joint.

The incudomalleal joint was visible and its synovial cavity had already formed (Figure 4). An important joint capsule formed by the direct continuation of the regional perichondrium could also be observed.

**Figure 5:**
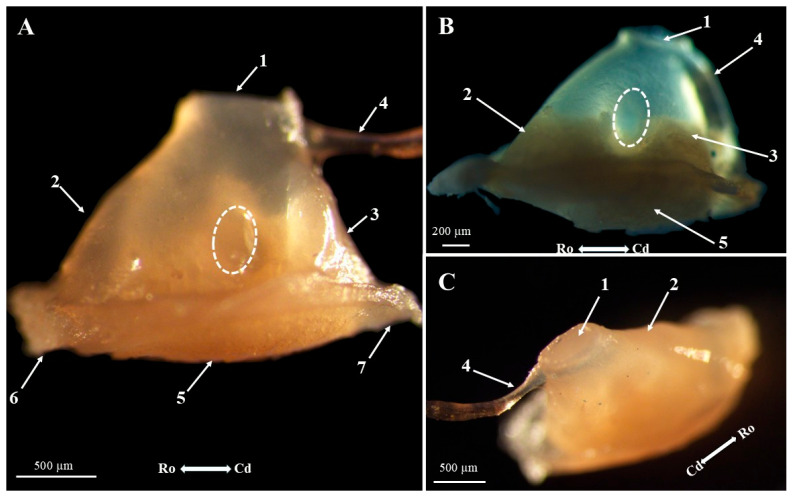
Stapes—anatomical features. (**A**). Right stapes, dorsal view; (**B**). right stapes, dorsal view; (**C**). right stapes, medial view. 1. Articular surface of the head of the stapes; 2. rostral crus of the stapes; 3. caudal crus of the stapes; 4. tendon of insertion of the stapedial muscle; 5. base of the stapes, vestibular surface; 6. rim of the footplate, anterior segment; 7. rim of the footplate, caudal segment; dotted circle—intercrusal foramen.

Macroscopical and morphometrical assessments revealed that the stapes (Figure 5) was the smallest auditory ossicle at the most medial aspect of the auditory chain. Overall, the stapes was triangular and allowed identification of the head of the stapes (*caput stapedis*), the base of the stapes (*basis stapedis*), the rostral crus (*crus rostrale*), and the caudal crus (*crus caudale*) (Figure 5).

Having a clearly cartilaginous architecture, the head of the stapes was covered by an ellipsoidal articular surface for association with the lenticular process of the long crus of the incus. It was also obvious that the central part of the articular surface had an excavated shape, probably having a direct correlation with the synovial sac of the incudostapedial joint (*articulatio incudostapedia*). The anterior surface of the head of the incus was slightly concave, but its caudal surface was almost straight. The tendon of insertion of the stapedial muscle (*m. stapedius*) was well developed and its attachment point on the caudal aspect of the head of the stapes could be identified in the large majority of our specimens; however, the stapes’ muscular process could not be confirmed (Figure 5). Morphological investigation did not identify any anatomical structure correlated with the presence of Paaw skeletal elements in the dog fetuses studied.

Medially, the head of the stapes was continued by two divergent crura of the stapes. The anterior crus ran in a rostro-medial direction and merged with the anterior segment of the tympanic surface of the stapedial footplate. A slightly anterior convexity could be observed at this level. The *crus caudale* was straight, ran in a medial direction, and merged with the caudal segment of the tympanic surface of the stapedial footplate (Figure 5). The caudal crus was shorter than the anterior crus in all our investigated specimens (Table 6). This feature explains the triangular shape of the stapes in dog fetuses.

The intercrusal foramen occurred as an oval-shaped space positioned between the two crura of the stapes. Because all the fetuses had the same gestational age, no variability of the intercrusal foramen was recorded.

The most medial segment of the stapes was the base, or footplate, which covered the vestibular fenestra. Its oval shape appeared to be a perfect match for the *fenestra vestibuli.* Consequently, the annular ligament (*lig. anulare stapedis*) was very short as observed histologically (Figure 5). Externally, the footplate margin is surrounded by a cartilaginous rim, thicker orally and thinner posteriorly.

**Figure 6:**
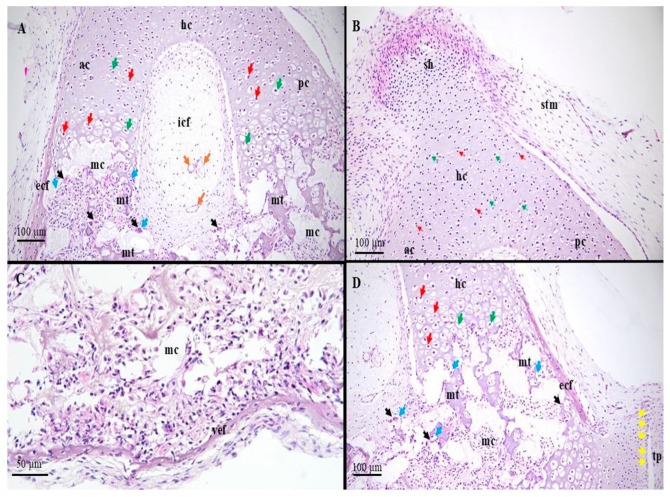
Histological features of the stapes in dog fetuses at 55–56 days of gestation. (**A**) anterior and caudal crura/H&E staining; (**B**) stapes head area/H&E staining; (**C**) stapes footplate area/H&E staining; (**D**) caudal crus and the stapedio-vestibular joint/H&E staining; ac—anterior crus; pc—posterior crus; mc—medullary cavity; mt—mineralized trabeculae; icf—intercrusal foramen; ecf—external cortical fascicle; vef—vestibular cortical fascicle; hc—hyaline cartilage; sh—head of the stape; stm—stapedial muscle tendon; tp—temporal bone, petrosal part; red arrows—chondrocyte; green arrows—condrocyte lacuna; black arrows—osteoclast; blue arrows—Howship’s lacunae; orange arrows—blood vessels; yellow arrows—anular ligament.

The histological architecture of the stapes (Figure 6) revealed that ossification had begun distally and involved both crura and the footplate.

In dog fetuses at 55–56 days of gestation, the distal ends of the anterior and posterior crura displayed an external mineralised cortical fascicle in each, but the inner fascicles showed large areas of interruption (Figure 6). Due to these gaps, the distal medullary cavities could communicate with the primitive middle ear space, allowing their contents to exit and come into contact with adjacent fetal mesenchymal tissues. Multiple mineralized endosteal trabeculae could be observed inside the medullary cavity for both crura. Ossification ceased in the middle third of the crura while the proximal end of the stapes maintained a typical cartilaginous appearance (Figure 6).

Osseous remodeling of the footplate had also been initiated in our specimens. The medullary cavity had a compartmentalized appearance, and mineralized endosteal trabeculae were visible at this level. In the investigated specimens, the auditory fascicle showed numerous interruptions, and communication with the tympanic cavity was also possible. The central segment of the vestibular cortical fascicle showed the first signs of ossification, whereas its extremities maintained their cartilaginous structure. The presence of a short annular ligament was noted (Figure 6).

## 4. Discussion

Morphological and morphometric assessments of the auditory ossicles have been performed on adult specimens of many mammalian species [6]. Until now, the fetal stages of these ossicles have been poorly investigated.

The first feature observed immediately after entering the tympanic bulla was a gelatinous mass that embedded all the structures found in the tympanic cavity. The presence of this gel-like liquid in these dog fetuses at 55–56 days of gestation was previously observed in 58-day-old Nellore sheep fetuses [36]. A similar tissue has also been reported in *Monodelphis domestica* specimens at 35 days of age [37]. The authors described this structure as a “loose mesenchyme”. It has been reported that in human fetuses, resorption typically begins in week 21 of fetal development and continues into childhood [38].

Although the entire cavity had a cartilaginous appearance and a gelatinous matrix surrounded the structures, this did not prevent us from identifying all components of the auditory chain in every specimen. The presence of all three ossicles is one of the most important features of all extant mammals [13] and in many other mammalian species [8,17,36,37,39].

The topographical position of the malleus in our specimens was consistent with the reported data for adult or fetal specimens [14,28,29,40,41,42,43,44]. In our investigated specimens, this ossicle was the largest and most laterally positioned, with one of its segments in direct contact with the tympanic membrane. Despite its cartilaginous appearance, the malleus in our specimens showed all three segments: the head, neck, and handle. Among all the described segments, the head of the malleus appears to have the greatest variability. In these specimens, the malleal head was oval and bore an articular surface for association with the incus on its caudo-medial surface. This oval head shape has been previously reported in other fetal specimens [6,14,29,45] and in many other adult specimens [46,47]. Other reported shapes include a globose malleal head in *Pantera pardus* [13], a spherical malleal head in mole-rats, or a bullet-shaped malleal head in caviomorph species [44,48,49].

According to morphometric assessments of the studied material, the malleus neck was short and difficult to identify in our specimens. A short neck of the malleus has also been reported in sheep and human fetuses [26,29,36,50]. These findings also mirror those documented in *Meles meles* [43], *Chlorocebus sabaeus* [44], and human newborns [4,26]. On the contrary, pig fetuses and New Zealand rabbits have been reported in the literature as significantly longer relatively [6,51]. Although the above-described segment was short and cartilaginous in all studied specimens, it carried all three processes described in the literature for adult dogs [52] and many other studied species [6,29,47].

In the specimens investigated, the anterior process was present and exhibited considerable variability. In some specimens, this entity was longer than the malleal handle (Table 4 and Table 7). It seems that the intraspecific variability we reported in dog fetuses has been observed for this process in other species. Similar features of the same process have been reported in human fetuses [45] and newborns of *Canis lupus* and *Didelphi* [13,53]. Previous studies describe the anterior process as a short and wide process in adult dogs, a very long structure in the white rhinoceros, a triangular structure in pig fetuses, or a rose-thorn-shaped structure in ruminants [6,18,54]. A complete lack of the anterior process was reported in lamb fetuses [29,36]. Comparing the morphological features reported by us in dog fetuses with those reported by [18] in adult dogs, we observe that in both dogs and humans the anterior process becomes shorter after birth [29] (Table 4 and Table 7).

The muscular process, part of the malleal neck, was present in all studied specimens and consisted of a conical projection on the medial side of the malleus, serving as the insertion point for the tensor tympani muscle. The topographical position noted here in dog fetuses appears to be similar to that reported in many other species [8,41,55]. Even if the overall shape of the muscular process in these fetuses was similar to that reported in adult dogs [18,46], the dorsal bend of this process was absent in the specimens studied here (probably linked to the lack of muscular activity of the tensor tympany in intrauterine life).

The topographical position of the chorda tympani groove reported in these fetal specimens confirms the anatomical position of the chorda tympani nerve previously mentioned in adult specimens [52]. A narrow bony canal for the chorda tympani nerve has also been corroborated as perforating the base of the muscular process in dog and wolf specimens [56]. Different topographical features have been reported in primates and small Indian mongooses, in which this process was observed on the inner surface of the *manubrium mallei* [6,44,57].

As reported for some wild carnivores [8,55,58], the lateral process of our specimens was shorter than the muscular and anterior processes. Although there is reported data hinting at the lateral process that can be used as a landmark between the two segments of the tympanic membrane (*pars flaccida* and *pars tensa*) [18,46], in our specimens, the direct contact between this process and the inner side of the tympanic membrane could not be confirmed.

In contrast to our findings, in sheep and pig fetuses, the lateral process of the manubrium mallei is the most dorsal part of the malleus and comes into contact with the mucosal layer of the tympanic membrane [6,29,36].

The curved shape of the manubrium mallei reported here is consistent with a previous description of the manubrium mallei in canids as having a “banana shape” [18]. Similar descriptions have also been provided for this shape in some wild carnivores [8,41,55,58,59].

The triangular shape of the proximal end of the manubrium mallei reported previously in pig fetuses, adult dog, wolf, red fox, badger, and goat [6,8,18,41,43,55,58,59] was similar to measurements observed here in fetal specimens. Conversely, a non-curved manubrium mallei has been reported in Nellore sheep fetuses [36].

Data interpretation of the calculated indices (Table 7 and Table 8) assists in assessing the tissues’ functionality. In this case, as puppies are born deaf and blind (so their auditory system is practically blocked and not fully developed) [60] the evolution and progression of metrically related indices may serve as possible relative indicators of the maturation stages of the sound transmission system.Specifically, the malleal indices may quantify lever mechanics and also may infer hearing specialization [6,44,53,61].

Due to the very limited availability of metrical data for common carnivores, a description of specific differences in shape and form, as well as fetal development over time, is highly constrained by this limited dataset. In fact, raw data for domestic dogs could not be found during the literature search for this project.

The closest published sets are those of the adult wolf [62] and foxes [9,59], and even for these species, only a limited number of measurements have been published, making it impossible to calculate all indices. The only sets allowing for comparison were the manubrial length index, reflecting the relative elongation of the manubrial segment, and the lever-arm efficiency in terms of mechanical advantage.

The manubrium index (Table 8) reflects in this very case an approximate 70–74% elongation of the total length in both adult wolf and fox specimens. As for the embryonic dog specimens, there is a large variation in the measurements, most likely due to the shortness of the anterior process. Probably, this is due to the later elongation of the process since the manubrium reaches its full length earlier in development.

The histological characteristics of the malleus observed in dog fetuses at 55–56 days of gestation are consistent with data reported from 16 to 21-week-old human fetuses [38]. Although in 66-day-old pig fetuses the highest proportion of mineralization has been reported in the manubrial area [6], in dog fetuses at 55–56 days of gestation, the neck area appears to be the initial site of ossification. The compartmentalized feature of the medullary cavity reported here in dog fetuses and previously reported in fetal and adult specimens [6,44,57] is accommodated within the malleal head area. Relatively dense osteoclastic activity was observed in this area (indicated by Howship’s lacunae and osteoclasts).

The morphological elements of the incus studied in our fetal specimens were similar to those previously listed in adult specimens [52]. Measurements of the incus in these fetal dogs substantiated previous similar findings, which describe a rectangular body and two caudal processes with classical similarity to biradicular molar tooth being very obvious [7,8,40].

The morphometrical assessments of the long and short crura of the incus revealed minimal differences in length (Table 5). These features observed in dog fetuses at 55–56 days of gestation were consistent with known reported data in carnivores and other species [7,8,40,58].

The presence of the lenticular process previously reported in canine adult specimens and other wild carnivores [52,58] was confirmed in all of our fetal specimens. According to sources, the lenticular bone is absent in sheep fetuses [36].

Given the very limited set of valid measurements (Table 5), the interpretation of these indices should be treated with caution. The calculated indices are listed in Table 9. These values primarily reflect the ossicle’s geometry and its mentioned role as a mechanical transducer.

Among these indices, the only comparative elements we can ascertain are those that compare the incudal index (Table 10). This is a shape-ratio index that reflects the transmission geometry and mechanical advantage based solely on the dimensions of the long and short processes. As other values are not available in the literature sources, our comparative perspective is limited only to this element. There is a significant range of values (Table 10), which also complicates the limited source data, making interpretation dubious.

Histological features of the second auditory bone in these dog fetuses at 55–56 days of gestation were similar to the reported data of 16-week-old human fetuses [38]. According to the authors mentioned, in the human fetus, the first sign of ossification occurs in the incus at the level of the long process after week 14 of fetal development. The structural difference reported here between the short and long incus processes are similar to that of previous investigations [6]. It is known that this process in human fetuses initially have a cartilaginous structure and its trabecular organization occurs approximately 8 weeks after the appearance of the first nucleus of ossification [38]. This difference in ossification pattern between the long and short processes of the incus has also been reported in adult human beings [57].

The presence of the incodomalleal joint with a well-developed synovial cavity and a defined joint capsule noted here substantiates the idea that this joint is one of the oldest joints of the body [24,44].

Characterized by high variability in human fetuses and newborns [45,63], the stapes were triangular in all our specimens.Due to its position at the level of the medial end of the auditory chain, it acts as a piston transmitting tympanic membrane vibration to the perilymphatic fluid that fills the membranous labyrinth [52]. The geometrical characterization of this third auditory bone is highly variable across mammalian species. Similar findings in our data have been reported in dogs, foxes, and wolves [8,58,59,64], as well as in horses and hamsters [55,59]. In pig fetuses, adult pigs, adult ruminants, and sheep fetuses, a rectangular stapes has been reported [6,29,46,47]. It seems that this special morphological aspect is directly related to the length of the stapedial crura [6] (Table 11).

The lack of the muscular tuberosity from the caudal surface of the canine fetal stapes was similar to the reported data in small and large ruminant fetuses [29,36] and in human fetuses [63]. The presence of a well-developed tuberosity on the caudal surface of the stapedial head in adult specimens has a positive correlation with the muscular activity of the stapedial muscle [14].

The lack of visible development of Paaw’s cartilages in these specimens validates previous assumptions that this structure grows later than the malleus, incus, and stapes [60,61]. According to other literature, this structure has never been reported in canids [18], but it has been reported in other mammalian species [60,62].

Comparative elements of the indices for the stapes (Table 12) are unfortunately not possible due to the lack of measurements in the few previously published references. The only valid reference point is the crural index, an indicator of possible asymmetry of the ossicle with slight implications for the understanding of vibrational mechanics [10,10,63]. Some minor differences compared to adult foxes and wolves suggest that the anterior process has not yet reached full development, whereas the caudal process is typically longer (Table 12).

The structural investigation of the most medial ear ossicles showed that, in dog fetuses at 55–56 days of gestation, ossification is likely initiated in the distal end region (the distal half of the crura and the base of the stapes). In contrast, the proximal segments (proximal half of the crura and the head of the stapes) maintained their cartilaginous features.

Our findings confirm previously reported data stating that this bone has three ossification centers: one for each crura and one at the level of the footplate [38]. Similar cartilaginous features of the proximal segments of the stapes have also been reported in 66-day-old pig fetuses [6]. Although the process of ossification has barely been initiated and only a small part of the stapes has been ossified, the presence of a compartmented medullary cavity could be confirmed in all specimens studied here. Similar findings have been reported in human fetuses [38,50]. The communication noted between the medullary cavities of the stapes crura and the fetal tympanic cavity has also been described in the literature and appears to result from an erosive process involving the internal cortical fascicles. These findings support the previously noted hematopoietic and immunologic roles of the marrow content that invades the fetal mesenchyme [38,64].

## 5. Conclusions

Despite this cartilaginous-like environment, all three ossicles and their segments are identifiable, and their overall anatomo-topographical pattern is consistent with adult and other fetal mammalian species.

The basic mammalian pattern of three-ossicle ossicular chain is already established in fetal dogs, with highly conserved anatomo-topographical relations and segmental morphology, yet with some notable species-specific and stage-dependent variations.

The malleus in dog fetuses is the largest and the most laterally placed ossicle with an oval head, a short neck, and a curved manubrium. The highest variability was observed at the level of the anterior process, whereas the lateral and muscular processes exhibit species-specific features.

The incus in dog fetal specimens is a rectangular piece with 2 processes of minimal length difference, similar to that found in other carnivora. The lenticular process is profiled in all studied specimens at this early stage and is linked to the incus by a synovial joint.

The stapes appears as a typical triangular shape, conforming to the standard descriptive elements. A notable feature is the reduced muscular process, which again suggests the absence of muscular activity during remodeling in this early stage.

The ossicles appear to ossify from distinct centers, but significant portions remain cartilaginous. From a histological perspective, ossification begins in specific regions of the ossicles (the neck of the maleus, the distal crura of the stapes and incus, and the stapedial footplate region), with medullary cavities already compartmentalized, showing some early osteoclast activity and communication with the surrounding mesenchymal spaces. These observations may also serve as an early sign of involvement of the bony spaces within the ossicles in the immunological and hematopoietic function.

Regardless of the limited number of available measurements, a preliminary attempt at functional characterization of these ossicles was performed using indices of the auditory ossicles. However, future studies utilizing larger metrical data sets are required to validate these findings.

## Figures and Tables

**Table 1:** Standardized measurements and indices for the malleus [35].

*X* axis	midpoint of the minimum neck width—the most noticeable point along the top of the head
*Y* axis	most inferior point of the short process and the manubrium
1 total length	tip of the manubrium to the top of the head
2 manubrium length	tip of the short process to the tip of the manubrium following *X* axis
3 manubrium M-L thickness	M-L thickness of the manubrium at mid manubrium length, perpendicular to *X* axis
4 manubrium arc depth	maximum depth of the curvature of the arc of the manubrium, following *X* axis
5 corpus length	tip of the head to the lower border of the manubrium following *X* axis
6 neck width	anterior and posterior borders of the neck
7 S-L head width	maximum distance between 2 parallel lines marking the widest points of the margin of the head, taken following the *X* axis
8 angle between axes	X-Y angle
manubrium/length index	(manubrium length/total length) × 100
manubrium robusticity index	(manubrium ML thickness/corpus length) × 100
manubrium/corpus index	(manubrium length/corpus length) × 100
corpus/length index	(corpus length/total length) × 100

**Table 2:** Standardized measurements and indices for the incus [35].

*X* axis	line that joins the most salient point along the anterior portion of the superior border of the body
*Y* axis	line that joins the tip of the long process to the most salient point along the superior border of the body
*Z* axis	line joining the tip of the long process to the most external point along the margin of the anterior facet
9 Short process length	maximum distance from the tip of the short process to the most salient point along the anterior portion of the superior border of the body, following *X* axis
10 Long process length	maximum distance from the tip of the long process to the most salient point along the superior border of the body
11 Functional length of the long process	perpendicular distance from the *Z* axis (rotational axis) to the tip of the long process
12 Arc depth of the long process	maximum depth of the arc along the long process measured from the plane defined by the lateral outmost point along the tip of the long process
13 Articular facet height	max height of the articular facet with the bone oriented along the rotational axis
14 Angle between the axes	angle formed by the *X* and *Y* axes
15 Interprocess length	maximum distance between the most salient points along the superior margin of the short process and the tip of the long process
16 Interprocess arc depth	maximum depth of the curvature between the short and long process tips
Incudal index	9/10 × 100
Long process index	11/10 × 100
Relative articular facet height	13/10 × 100

**Table 3:** Standardized measurements and indices for the stapes [35].

*X* axis	Line joining the antero-superior corner of the footplate and the tip of the head
*Y* axis	Line joining the posterior-superior corner of the footplate and the tip of the head
*Z* axis	Line joining the most inferior points along the footplate margin anteriorly and posteriorly
19 Total height of the stapes	Maximum height from the lower margin of the footplate to the tip of the head, perpendicular to the *Z* axis
20 Head height	Minimum distance between the superior margin of the obturator foramen and the top of the head, taken perpendicular to the *Z* axis
21 Obturator foramen height	Maximum height of the obturator foramen taken perpendicular to the *Z* axis
22 Obturator foramen width	Maximum width of the obturator foramen taken parallel to the *Z* axis
23 Maximum width of the crura	Maximum width across the anterior and posterior crurae, taken on the external aspect and parallel to the *Z* axis
24 Posterior crus length	Maximum distance from the posterior-superior corner of the footplate to the tip of the head, following *Y* axis
25 Posterior crus arc depth	Maximum depth of the curvature of the posterior crus taken parallel to the *Y* axis
26 Anterior crus length	Maximum distance from the anterio-superior corner of the footplate to the tip of the head following *X* axis
27 Anterior crus arc depth	Maximum depth of the curvature of the anterior crus taken parallel to the *X* axis
28 Angle A	Angle between the anterior and posterior crurae or between the *X* and *Y* axes
29 Angle B	Angle between the anterior crus and the footplate or between the *X* and *Z* axes
30 Angle C	Angle between the posterior crus and the footplate between *Y* and *Z* axes
31 Footplate length	Maximum length of the footplate
32 Footplate width	Maximum width of the footplate
33 Footplate area	Measured area of the footplate
Stapedial index	31/19 × 100
Relative head height	20/19 × 100
Obturator foramen index	21/22 × 100
Footplate index	31/32 × 100
Crural index	36/24 × 100

**Table 4:** Metric dimensions for the malleus in investigated dog individuals.

Measurement Number	S2R	S31R	S41	S42
1	4.93 mm (n = 3)	6.18 mm (n = 7)	5.56 mm (n = 2)	4.97 mm (n = 3)
2	5.15 mm (n = 3)		7.23 mm (n = 2)	7.8 mm (n = 2)
3	0.63 mm (n = 3)	0.75 mm (n = 4)	0.70 mm (n = 2)	0.53 mm (n = 3)
5	1.42 mm (n = 3)	1.06 mm (n = 1)	2.68 mm (n = 2)	2.13 mm (n = 3)
6	3.04 mm (n = 3)		3.05 mm (n = 2)	2.39 mm (n = 3)
7	2.51 mm (n = 3)	2.22 mm (n = 6)	3.85 mm (n = 5)	3.28 mm (n = 3)

**Table 5:** Metric dimensions for the incus in investigated dog individuals.

Measurement Number	S2R	S31R	S41
9	3.07 mm (n = 1)	3.04 mm (n = 1)	
10	3.14 mm (n = 1)_	3.29 mm (n = 1)	
11	1.84 mm (n = 1)	1.69 mm (n = 1)	
13	1.4 mm (n = 1)	1.83 mm (n = 1)	2.21 mm (n = 1)
14	53	52.6	
15	2.52 mm (n = 1)	2.46 mm (n = 1)	
16	0.71 mm (n = 1)	0.69 mm (n = 1)	

**Table 6:** Metric dimensions of the stapes in the investigated dogs.

Measurement Number	S2R	S31R	S41
19	1.61 mm (n = 2)		2.09 mm (n = 5)
20			0.77 mm (n = 2)
21			1.12 mm (n = 2)
22	0.67 mm (n = 1)		0.89 mm (n = 2)
23	1.47 mm (n = 2)		1.9 mm (n = 3)
24	2.1 mm (n = 1)		2.29 mm (n = 2)
25			0.43 mm (n = 3)
26	1.76 mm (n = 1)		2.14 mm (n = 2)
27			0.48 mm (n = 2)
31	1.99 mm (n = 3)	2.15 mm (n = 4)	2.066 mm (n = 5)
32	1.07 mm (n = 1)	0.81 mm (n = 2)	0.79 mm (n = 1)
33	1.484 mm^2^ (n = 1)	1.23 mm^2^ (n = 2)	1.191 mm^2^ (n = 1)

**Table 7:** Variability of indices of the malleus in fetal dog specimens.

Index Malleus	Value (Average/Variation)
manubrium/length index	130.4 (+/−22.64)
manubrium robusticity index	9.60 (+/−2.37)
manubrium/corpus index	349.32 (+/−66.38)
lenght index	37.36 (+/−11.95)

**Table 8:** Calculated values for the manubrial index.

Manubrial Length Index	Value (Average/Variation)
Embrionic dog	130.4 (+/−22.64)
Wolf (adult)	73.08 (+/−1)
Fox (adult)	72.69 (+/−1.56)

**Table 9:** Variance indices of the incus.

Index	Value (Average/Variation)
Incudal index	95.08(+/−3.79)
Long process index	54.98 (+/−5.11)
Relative articular facet height	50.1(+/−7.80)

**Table 10:** Comparative recalculated values for the incudal index.

Manubrial Length Index	Value (Average/Variation)
Embrionic dog	95.08 (+/−3.7)
Wolf (adult)	87.54 (+/−1.14)
Fox (adult)	72.20 (+/−1.56) [58] 96.8 (+/−1.58) [8]

**Table 11:** Variance indices of the stapes.

Index	Value (Average/Variation)
Stapedial index	93.14 (+/−42.94)
Relative head height	33.52 (+/−1.32)
Obturator foramen index	126.97 (+/−0.12)
Footplate index	19.90
Crural index	90.14 (+/−9.32)

**Table 12:** Comparative recalculated values for the stapedial crural index.

Crural Index	Value (Average/Variation)
Embrionic dog	90.14 (+/−9.32)
Wolf (adult)	110.29 (+/−0.19)
Fox (adult)	102.42 (+/−0.72) 120.3 (+/−3.81)

## Data Availability

The original contributions presented in this study are included in the article. Further inquiries can be directed to the corresponding author.
